# Tumour blood flow for prediction of human prostate cancer aggressiveness: a study with Rubidium-82 PET, MRI and Na^+^/K^+^-ATPase-density

**DOI:** 10.1007/s00259-020-04998-2

**Published:** 2020-08-18

**Authors:** Mads Ryø Jochumsen, Jens Sörensen, Bodil Ginnerup Pedersen, Jens Randel Nyengaard, Søren Rasmus Palmelund Krag, Jørgen Frøkiær, Michael Borre, Kirsten Bouchelouche, Lars Poulsen Tolbod

**Affiliations:** 1 Department of Nuclear Medicine and PET-Centre, Aarhus University Hospital, Aarhus, Denmark; 2 Department of Clinical Medicine, Aarhus University, Aarhus, Denmark; 3 Department of Radiology, Aarhus University Hospital, Aarhus, Denmark; 4 Core Centre for Molecular Morphology, Section for Stereology and Microscopy, Centre for Stochastic Geometry and Advanced Bioimaging, Aarhus University, Aarhus, Denmark; 5 Department of Pathology, Aarhus University Hospital, Aarhus, Denmark; 6 Department of Urology, Aarhus University Hospital, Aarhus, Denmark

**Keywords:** Tumour blood flow, Prostate cancer, Rubidium-82, Na^+^/K^+^-ATPase, ISUP grade group

## Abstract

**Purpose:**

Tumour blood flow (TBF) is a crucial determinant of cancer growth. Recently, we validated Rubidium-82 (^82^Rb) positron emission tomography (PET) for TBF measurement in prostate cancer (PCa) and found TBF and cancer aggressiveness positively correlated. The aims of the present study were to determine the ability of TBF for separating significant from insignificant PCa and to examine the relation to underlying Na^+^/K^+^-ATPase density, which is relevant as ^82^Rb is transported intracellularly via the Na^+^/K^+^-ATPase.

**Methods:**

One hundred and two patients were included for pelvic ^82^Rb PET scan prior to magnetic resonance imaging (MRI)-guided prostate biopsy. Findings constituted 100 PCa lesions (86 patients) and 25 benign lesions (16 patients). Tumours were defined on MRI and transferred to ^82^Rb PET for TBF measurement. Immunohistochemical Na^+^/K^+^-ATPase staining was subsequently performed on biopsies.

**Results:**

TBF was the superior predictor (rho = 0.68, *p* < 0.0001, inflammatory lesions excluded) of MRI-guided biopsy grade group (GG) over lowest apparent diffusion coefficient (ADC) value (rho = −0.23, *p* = 0.01), independent of ADC value and tumour volume (*p* < 0.0001). PET could separate GG-2-5 from GG-1 and benign lesions with an area under the curve (AUC), sensitivity, and specificity of 0.79, 96%, and 59%, respectively. For separating GG-3-5 from GG-1-2 and benign lesions the AUC, sensitivity, and specificity were 0.82, 95%, and 63%, respectively. Na^+^/K^+^-ATPase density per PCa cell profile was 38% lower compared with that of the benign prostate cell profiles. Neither cell density nor Na^+^/K^+^-ATPase density determined tumour ^82^Rb uptake.

**Conclusion:**

TBF is an independent predictor of PCa aggressiveness and deserves more attention, as it may be valuable in separating clinically significant from insignificant PCa.

**Electronic supplementary material:**

The online version of this article (10.1007/s00259-020-04998-2) contains supplementary material, which is available to authorized users.

## Introduction

As a cornerstone of cancer growth, tumour blood flow (TBF) has been studied and used for characterization of a range of tumours, including prostate cancer (PCa) [1]. Recently, Cristel et al. showed a substantial improvement of the positive predictive value of multiparametric (mp) magnetic resonance imaging (MRI) for primary local staging of PCa by quantitative pharmacokinetic analysis of the dynamic contrast enhanced (DCE) series and applying a K_trans_ cut-off to PIRADS 3 lesions [2]. However, the role of DCE MRI in PCa imaging is currently under heavy discussion, as bi-parametric MRI (T2w and diffusion-weighted sequences including apparent diffusion coefficient (ADC) map) is gaining foothold [3]. Several recent studies found only slight additional value of the qualitative PIRADS DCE analysis compared with bi-parametric MRI for highly experienced readers [3, 4], while others described larger additional value of DCE [5]. Removing DCE from the protocol can reduce examination time and cost, and remove the contrast agent-related morbidity, which could be helpful as more and more prostate MRI scans are requested [3].

Rubidium-82 (^82^Rb) functions as a blood flow tracer for positron emission tomography (PET), as the ^82^Rb uptake in metabolically active tissue is proportional to the actual blood flow. ^82^Rb PET is used in clinical routine for myocardial blood flow measurement at many PET centres worldwide, especially in the USA, as FDA approves it for reimbursement. Increased ^82^Rb uptake has been reported in various cancers, including breast cancer [6], lung cancer [7], renal cell carcinoma [8], and neuroendocrine tumours [9]. Recently, we validated ^82^Rb PET/computed tomography (CT) for TBF measurement in PCa and demonstrated that ^82^Rb uptake in PCa was significantly higher than in healthy prostate tissue [10, 11].

Due to close proximity in the periodic table of elements, ^82^Rb is actually a potassium analogue with parallel cellular uptake and renal excretion [12, 13], and was consequently used as a marker of potassium in physiological transport studies [14–16]. During our studies, enhanced renal rubidium excretion was seen on ^82^Rb PET in one-tenth of the patients, and roughly half of these patients had either hypokalaemia or received thiazide treatment [17]. The active transport of rubidium and potassium into cells is conducted predominantly via the Na^+^/K^+^-adenosine triphosphatase (Na^+^/K^+^-ATPase) also known as the Na^+^/K^+^-pump [18, 19]. Na^+^/K^+^-ATPase activity is of therapeutic interest as the Na^+^/K^+^-ATPase inhibitory effects of digitalis glycosides have shown anti-proliferative potential in PCa [20–22].

In our previous studies on PCa, we found a positive correlation between cancer aggressiveness and TBF, measured with both [^15^O]H_2_O and ^82^Rb PET [10, 23]. Similar results were found when studying K_1_ influx of other tracers including [^18^F]flourocholine [24, 25], [^11^C]donepezil [26], and [^11^C]acetate [27]. Consequently, quantitative TBF measurement may be promising in PCa imaging. However, previous studies included relatively small cohorts. Hence, the main purpose of the present study was to determine the correlation between TBF and PCa aggressiveness in a large prospective clinical study. To further evaluate the underlying biology of ^82^Rb uptake in PCa, we examined to which extend the ^82^Rb uptake reflects Na^+^/K^+^-ATPase density.

## Materials and methods

### Patient population

One hundred and two patients scheduled for MRI-guided prostate biopsies were prospectively included in the study. The sole inclusion criterion was a scheduled MRI-guided prostate biopsy. Exclusion criteria were other known pelvic cancers, previous PCa treatment, or alloplastic hip. The patients underwent dynamic pelvic ^82^Rb-PET/CT prior to MRI-guided biopsy. A 3-Tesla mpMRI with at least one PIRADS 3–5 lesion was available for all participants prior to inclusion. MRI-guided prostate biopsies were scored according to International Society of Urological Pathology (ISUP) grade group (GG). Inflammation in biopsies was registered when described in the clinical pathology report. Immunohistochemically (IHC) Na+/K + -ATPase staining and cell profile counting were subsequently performed on MRI-guided biopsies.

The institutional review board (Central Denmark Region Committees on Health Research Ethics) approved the study, and all subjects signed a written informed consent.

### Imaging

Most ^82^Rb PET/CT scans (93/102) were performed on a GE Discovery MI Digital Ready PET/CT (GE Healthcare, Waukesha, Wisconsin, USA), while nine scans were performed on GE Discovery MI (5 ring) solid-state detector PET/CT. Details of the scan and reconstruction protocols have previously been described in details [10]. In short, a low-dose CT was used for attenuation correction. Eight-minute scans were performed in list mode. The static image series (3- to 7-min post-injection) were used for SUV analysis. Reconstruction of the PET images was performed using the VuePointFX reconstruction algorithm with all common corrections applied. At scan start, a bolus injection of ^82^RbCl (1110 MBq) was performed by the CardioGen-82 generator infusion system (Bracco, Monroe Township, New Jersey, USA).

MpMRI scans were performed according to PIRADS v2.1 minimum protocol [28] with high-resolution T2w imaging of the prostate in sagittal, axial, and coronal plane, combined with DWI (*b* values 50, 400, 800) in all patients and T1w DCE scan with a temporal resolution of 3 s in 101 patients (124 lesions). ADC map and b-1400 images were calculated based on the DWI sequences. MpMRI scans were performed on 3-T platform Siemens Skyra (Siemens, Erlangen, Germany) before and after injection of Dotarem 2 ml/kg body weight (Dotarem, gadoterate meglumine 0.5 mmol/ml, ViCare Medical, Birkerød, Denmark).

Cancer suspicious lesions were identified using a dedicated prostate software (ProCAD, Nijmegen, The Netherlands), classified according to the PIRADS classification v2, and biopsied in-bore MRI-guided.

### Image analysis

The ^82^Rb-PET/CT scans were manually co-registered with the T2-weighted sequence of the mpMRI using the CT as reference. The tumour volume of interest (VOI) was drawn directly on the MRI scan and subsequently transferred to the ^82^Rb-PET/CT scan for TBF measurement. Image registration and VOI analysis, including tumour volume measurements, were performed with Hermes Hybrid Viewer (Hermes Medical Solutions, Stockholm, Sweden).

### Immunohistochemistry

MRI-guided prostate biopsies from 100 patients were available for immunohistochemical (IHC) Na^+^/K^+^-ATPase staining. One patient (patient 35) did not give informed consent to the IHC staining, and from patient 78, there was no tissue left for analysis. These two patients were excluded from IHC.

The presence of α1- and α2-subtypes of Na^+^/K^+^-ATPase was initially tested in a subgroup, revealing abundant expression of the α1-isoform, and no expression of the α2-isoform in accordance with existing literature [29]. Hence the α1-Na^+^/K^+^-ATPase antibody was utilized in the study.

Negative and positive controls were performed for each staining batch. For positive controls, we used human prostate tissue and multi-organ mouse tissue.

IHC staining was performed in accordance with the OptiView DAB IHC v6 procedure on a BenchMark ULTRA IHC/ISH Staining Module (Ventana Medical Systems, Tucson, Arizona, USA). The analyses were performed on 3-μm thick sections from the paraffin-embedded prostate tissue.

After deparaffination, antigen retrieval was performed using Cell Conditioning Solution (CC1) with a pH ~ 9 (Ventana Medical Systems, Tucson, Arizona, USA). The α-1 subtype of Na^+^/K^+^-ATPase was labelled using α5-s as primary antibody (15.13 μg/ml) (DSHB, Iowa City, Iowa, USA) and OptiView as secondary antibody (goat anti-mouse) on Ventana BenchMark (Ventana Medical Systems, Tucson, Arizona, USA). Detection of the secondary antibodies was achieved using 3.3′-diaminobenzidin (DAB). IHC slides were counterstained with haematoxylin. The primary antibody used for IHC of the α2-isoform was 16836-1-AP (1:1000) (Proteintech, Rosemont, Illinois, USA) and the secondary antibody used was P0448 goat anti-rabbit (Dako Denmark A/S, Glostrup, Denmark).

After IHC, the slides were digitally scanned on NDP scanner and analysed in NDP.View2 (Hamamatsu Photonics K.K, Hamamatsu City, Japan). An expert pathologist assessed all biopsies and defined areas of cancer and non-malignant areas. Non-malignant areas were defined as either (1) benign tissue in a benign biopsy or (2) normal tissue area near the tumour area in biopsies with cancer.

Randomly located, 2D unbiased counting frames of 0.01 mm^2^ were used to sample cell nuclei profiles from representative malignant areas, representative non-malignant areas (including potential loose connective tissue), and areas with highest gathering of malignant and non-malignant glands (not in loose connective tissue), respectively. Cell profile density was calculated as follows (cell profiles/mm^2^): $\kern0.5em \frac{\ \mathrm{total}\ \mathrm{cell}\ \mathrm{profiles}\ \mathrm{counted}}{\mathrm{area}\ \mathrm{of}\ \mathrm{counting}\ \mathrm{frame}\bullet \mathrm{number}\ \mathrm{of}\ \mathrm{counting}\ \mathrm{frame}\mathrm{s}.}$

Quantification of the α1-Na^+^/K^+^-ATPase antibody staining intensity was performed using H-DAB colour-deconvolution function in ImageJ [30]. The brown Colour_2 image was quantified after calibrating the colours to uncalibrated OPTICAL DENSITY. The threshold used for quantification of the malignant and non-malignant areas was 0; 2.71, which include the entire region of interest (all area). For quantification of glands only, a 0.15; 2.71 threshold was used, as it almost exclusively includes the glandular structures within the given region of interest. Na^+^/K^+^-ATPase optical density per cell profile was estimated as follows: $\frac{\mathrm{Na}+/\mathrm{K}+-\mathrm{ATPase}\ \mathrm{optical}\ \mathrm{density}/\mathrm{mm}2}{\mathrm{cell}\ \mathrm{profiles}/\mathrm{mm}2}$

### Statistical analysis

Data were tested for normality using QQ-plots and histograms. Normally distributed data are reported as mean ± standard deviation and non-normally distributed data are reported as median with range and log-transformed in parametric analysis. *P* values < 0.05 were considered statistically significant.

Spearman’s rank correlation was used for analysis of correlations involving ISUP GG, which is an ordinal scale. For correlation analysis, GG = 0 was used for benign lesions. Pearson’s correlations were used for continuous variables. Univariate and multivariate linear regression analysis were applied for quantification of the differences in blood flow between GG groups and for correction for tumour volume and lowest ADC value.

As the non-malignant and malignant areas analysed for Na^+^/K^+^-ATPase originated from the same biopsies, a paired *t* test for difference in means was applied on normally distributed data and linear regression analysis for quantifying relative differences.

All data were collected and managed using REDCap (Vanderbilt University Medical Center, Nashville, Tennessee, USA) electronic data capture tools, hosted at Aarhus University [31]. Analysis was performed in STATA version 15.1 (StataCorp LLC, College Station, Texas, USA).

## Results

In total, 102 patients were included, scanned, and biopsied. All patients underwent ^82^Rb PET/CT within 1 week prior to in-bore MRI-guided biopsy, and, for most patients on the same date. One hundred twenty-six lesions were identified on MRI, one PIRADS 2 lesion, two PIRADS 3 lesions, eighty-one PIRADS 4 lesions, and forty-two PIRADS 5 lesions. Eighty-nine lesions were located in the peripheral zone and 37 in the transitional zone. PCa was found in 100 lesions from 86 patients. MRI-guided biopsy ISUP GG was available in 99 lesions from 85 patients, as one lesion contained too sparse material for Gleason grading. Twenty-five lesions from 16 participants were benign, and one biopsy was non-representative. Trans-rectal ultrasound (TRUS) biopsies had been performed in 98 patients prior to inclusion in the study. Median time from TRUS biopsy to ^82^Rb scan was 174 days, range [30; 3582]; amongst these, only three patients with less than 8 weeks interval, and hence previous biopsies, were not expected to influence the TBF measurement.

### Tumour blood flow predicts cancer aggressiveness

**Fig. 1:**
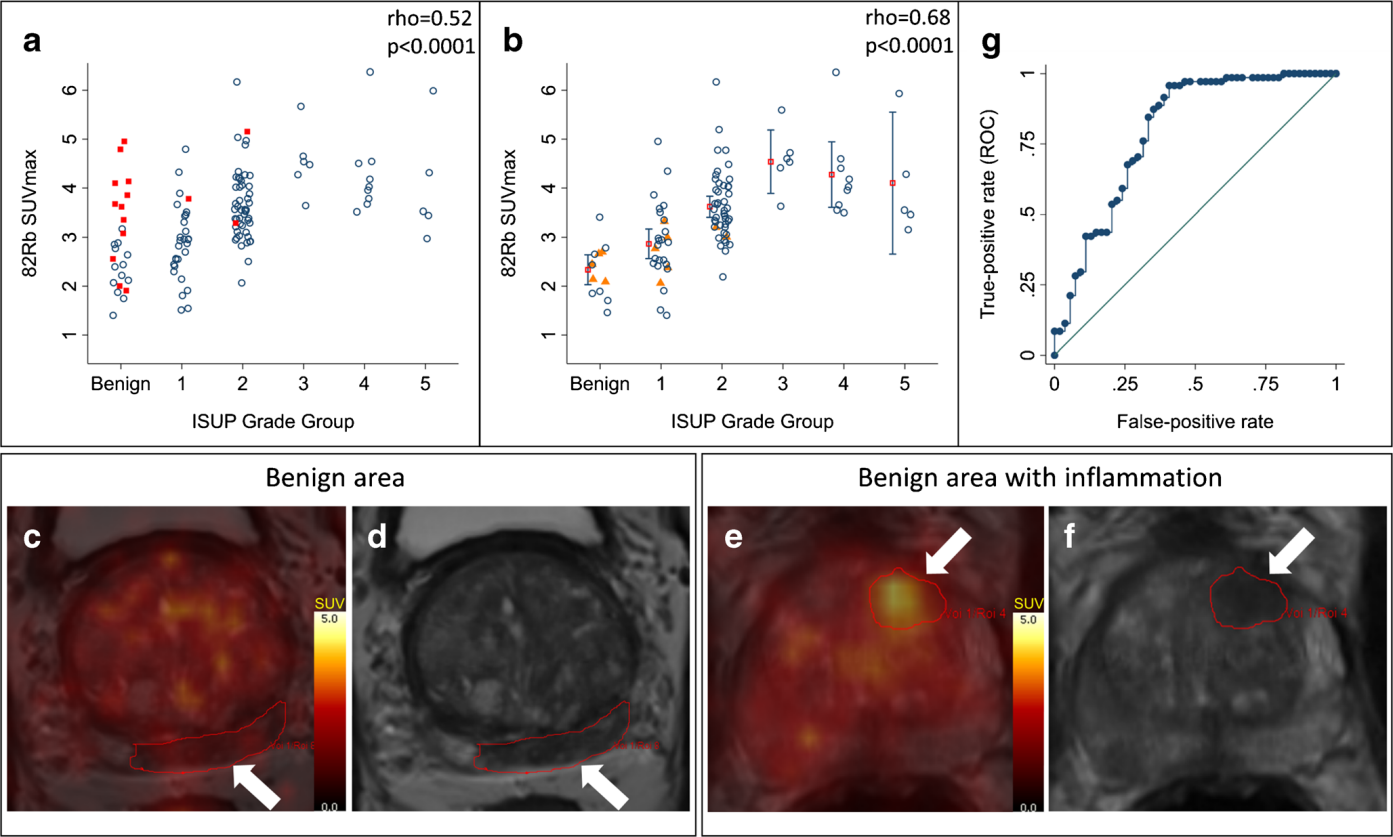
**a** Plot of all lesions; red squares are inflammatory lesions. **b** Plot when inflammation are excluded; orange triangles are lesions scored DCE negative in PIRADS, bars are mean (red square) with confidence interval (whiskers). **c** and **d** Fused ^82^Rb PET/MRI and t2w MRI, respectively, of a benign lesion without inflammation with low blood flow. **e** and **f** Fused ^82^Rb PET/MRI and t2w MRI, respectively, of a benign lesion with inflammation on pathology, with high blood flow. **g** ROC curve for ^82^Rb PET to differentiate significant cancer (ISUP GG-2-5) from insignificant findings (benign and GG-1); all lesions included

Blood flow measurements along with correlations are provided in Table 1. SUVmax performed slightly better than SUVmean and SUVpeak on Spearman’s rank correlation; besides SUVmax is less sensitive to VOI drawing methods, and therefore, SUVmax was primarily used in the following analyses and discussion. SUVmax and ISUP GG were moderately correlated (rho = 0.52, *p* < 0.0001, *n* = 124). SUVmax and ISUP GG including benign biopsies are plotted in Fig. 1a, but it is clear that benign inflammatory lesions (red squares) had significantly higher blood flow (3.53 [2.92; 4.14]) than benign lesions without inflammation (2.34 [2.03; 2.64]) (*p* = 0.001). When lesions with inflammation detected by the pathologist were excluded, including three inflammatory cancer lesions (Fig. 1b), the correlation improved (rho = 0.68, *p* < 0.0001, *n* = 109). Correlations for cancer only were rho = 0.56, *p* < 0.0001, and *n* = 99. A representative low flow benign lesion without inflammation is shown in Fig. 1c and d, and a high flow inflammatory benign lesion is illustrated in Fig. 1e and f.

**Table 1:** ^82^Rb lesion uptake for different groups of patients and correlations between ^82^Rb uptake and ISUP Grade Group (GG)

	Benign (*n* = 13)	Benign + inflammation (*n* = 12)	ISUP Grade Group					Correlations		
			1 (*n* = 29)	2 (*n* = 50)	3 (*n* = 6)	4 (*n* = 9)	5 (*n* = 5)	ISUP GG (*n* = 99)	ISUP GG* (*n* = 124)	ISUP GG** (*n* = 109)
SUVmax	2.34 ± 0.51	3.53 ± 0 .96	2.90 ± 0.78	3.65 ± 0 .73	4.54 ± 0 .62	4.28 ± 0 .87	4.10 ± 1.17	rho = 0.56 *p* < 0.0001	rho = 0.52 *p* < 0.0001	rho = 0.68 *p* < 0.0001
SUVmean	1.79 ± 0.44	2.45 ± 0.69	1.88 ± 0.48	2.28 ± 0.48	3.00 ± 0.39	2.81 ± 0.41	2.38 ± 0.43	rho = 0.53 *p* < 0.0001	rho = 0.41 *p* < 0.0001	rho = 0.56 *p* < 0.0001
SUVpeak	1.99 ± 0.53	2.76 ± 0.71	2.32 ± 0.56	2.74 ± 0.57	3.47 ± 0.53	3.21 ± 0.60	3.15 ± 0.76	rho = 0.49 *p* < 0.0001	rho = 0.44 *p* < 0.0001	rho = 0.58 *p* < 0.0001
Lowest ADC	811 ± 131	760 ± 193	752 ± 182	666 ± 178	615 ± 149	721 ± 228	757 ± 224	rho = − 0.13 *p* = 0.21	rho = − 0.23 *p* = 0.01	rho = − 0.22 *p* = 0.02

*GG = 0 was used for benign lesions, all lesions included. **GG = 0 was used for benign lesions, inflammatory lesions excluded

Linear regression analysis revealed a mean increase of 0.76 with 95% CI [0.56; 0.95] in ISUP GG per increase in SUVmax, when inflammatory biopsies are excluded (*p* < 0.0001) and 0.62 with 95% CI [0.41; 0.83] with inflammatory lesions included (*p* < 0.0001). Correction for tumour volume and ADC on multivariate linear regression did not substantially change the coefficients: 0.79 [0.57; 1.02], (*p* < 0.0001); and 0.62 [0.38; 0.86], (*p* < 0.0001), respectively.

Mean ^82^Rb SUVmax in low-risk PCa (ISUP GG-1) of 2.90 with 95% CI [2.60; 3.19] was significantly lower than in both intermediate risk (ISUP GG-2-3) 3.74 [3.54; 3.95] and high-risk (ISUP GG-4-5) PCa 4.21 [3.67; 4.76] (*p* < 0.001), when including all cancer lesions.

We performed an analysis with subdivision of ISUP GG2 with a group 2a with 5% or less Gleason 4, a group 2b with between 5 and 40%, and a group 2c with 40–49% Gleason 4 as discussed in previous papers [32, 33]. However, there was no difference in means between SUVmax of group 2a, 2b, and 2c, and the subdivision did not change the overall correlation with SUVmax with either inflammatory lesions excluded (rho = 0.66, *p* < 0.0001) or included (rho = 0.51, *p* < 0.0001).

With an AUC of 0.79 **(**Fig. 1g**)**, ^82^Rb PET could separate ISUP GG-2-5 from ISUP GG-1 and benign biopsies with a sensitivity of 96% and a specificity of 59% with 2.92 as SUVmax cut-off. For separating ISUP GG-3-5 from ISUP GG-1-2 and benign biopsies, the AUC was 0.82, with a sensitivity of 95% and a specificity of 63% with 3.48 as SUVmax cut-off. Data from all lesions are used for the included receiver operating characteristics (ROC) analysis, as there was no way of knowing a priori whether the detected high uptake was true PCa or false positive, due to inflammation. All ROC calculations are found in Supplementary Table 1 and 2.

The scans performed on GE Discovery MI Digital Ready PET/CT and GE Discovery MI (5 ring) PET/CT were very similar in SUV values and correlation to GG, so all scans were equally included in all analysis.

### Lowest ADC value and DCE as predictors of cancer aggressiveness

We found a weak negative correlation between lowest ADC value and ISUP GG (rho = − 0.23, *p* = 0.01, *n* = 124), benign lesions included **(**Table 1**)**. Correlation for cancer only was rho = − 0.13, *p* = 0.21, *n* = 99. ROC AUC for lowest ADC value for separating ISUP GG > 1 and ISUP GG > 2 was 0.64 and 0.56, respectively (Supplementary Table 1 and 2).

All lesions were classified based on mpMRI and according to PIRADS v2, where the lesions are classified as plus or minus for the presence or absence of focal, earlier, or contemporary enhancement compared with adjacent normal prostate tissue. Only 12 lesions were DCE negative. These lesions were all relatively low in ^82^Rb TBF (mean SUVmax 2.66 ± 0.47) and are marked as orange triangles in Fig. 1b. Five of the DCE negative lesions were benign (mean SUVmax 2.44 ± 0.30), five were ISUP GG-1 (mean SUVmax 2.68 ± 0.57), two were ISUP GG-2 (mean SUVmax 3.14 ± 0.21), and none was ISUP GG-3-5. All of the inflammatory lesions were DCE positive (12 benign lesions and 3 PCa lesions).

### Na+/K+-ATPase optical density in cancer versus normal prostate tissue

**Fig. 2:**
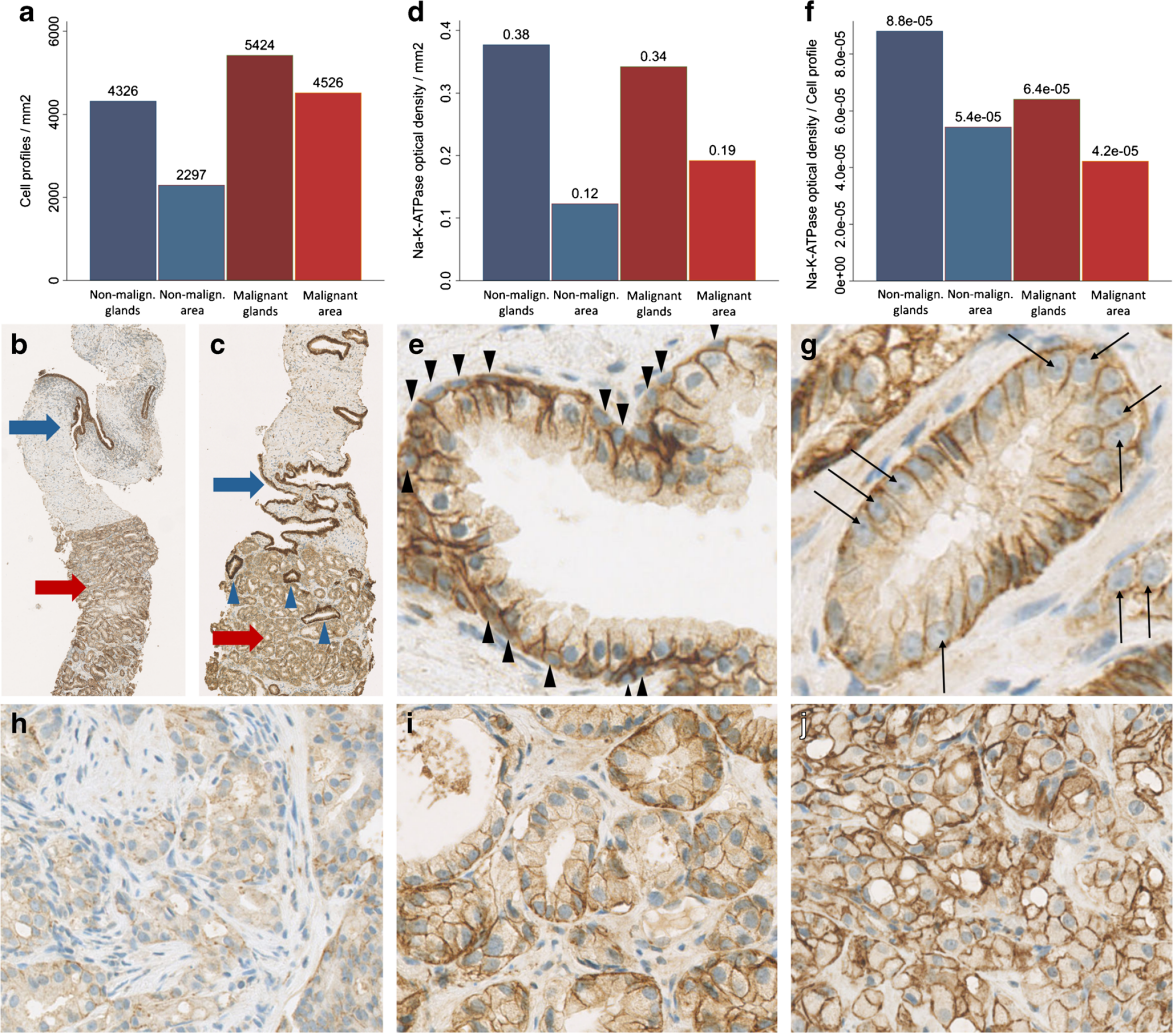
Na^+^/K^+^-ATPase immunohistochemistry and cell profile counting. **a** Bar chart showing cell profile density for non-malignant glands, non-malignant area, malignant glands, and malignant area, respectively. **b** Shows that malignant (red arrow) and non-malignant area (blue arrow) are distinguished in low magnification in most biopsies. **c** Shows a malignant area (red arrow) with solitary non-malignant glands (blue arrowheads). **d** Bar chart showing Na^+^/K^+^-ATPase optical density per area. **e** Illustrates the double cell layer of non-malignant glands; arrowheads point out basal cell layer. **f** Bar chart over Na^+^/K^+^-ATPase optical density per cell. **g** (black arrows) Malignant cells have larger nuclei with nucleoli and no basal cells (ISUP GG-1). **h** Shows GG-4 cancer with substantial loss of Na^+^/K^+^-ATPase optical density. **i** GG-2 cancer, **j** GG-5

Data from IHC and cell density measurements are provided in Table 2. Mean cell density (cell profiles/mm^2^) are 24% (1.24 with 95% CI [1.17; 1.33]) higher for malignant glands than for non-malignant glands, and 96% higher (1.96 with 95% CI [1.83; 2.09]) (*p* < 0.0001) for the entire malignant area than for the entire non-malignant area **(**Fig. 2a**)**.

**Table 2:** Results of Na^+^/K^+^-ATPase immunohistochemistry and cell profile counting

	Na+/K+-ATPase optical density *Intensity/mm*^*2*^	Cell profile density *Cell profiles/mm*^*2*^	Na+/K+-ATPase optical density per cell profile *Intensity/cell profile*
Non-malignant glands	0.38 ± 0.036	4326 ± 967	8.8E-5 [4.1E-5; 15.8E-5]
Non-malignant area	0.12 ± 0.031	2297 ± 553	5.4E-5 [2.8E-5; 9.8E-5]
Malignant glands	0.34 ± 0.053	5424 ± 1432	6.4E-5 [3.2E-5; 13.9E-5]
Malignant area	0.19 ± 0.047	4526 ± 1205	4.2E-5 [2.0E-5; 10.8E-5]

The malignant and non-malignant areas were easily separated in most biopsies at low magnification (Fig. 2b). Upon visual evaluation of a malignant area with solitary non-malignant glands (Fig. 2c, blue arrows), the non-malignant glands clearly have higher Na^+^/K^+^-ATPase expression than malignant glands.

Figure 2d shows the measured IHC colour intensity, which is equivalent to Na^+^/K^+^-ATPase optical density. Non-malignant glands (0.38 ± 0.036) have 12% higher Na^+^/K^+^-ATPase optical density than malignant glands (0.34 ± 0.053) (*p* < 0.0001), and on the contrary, the entire malignant areas (0.19 ± 0.047) has 58% higher Na^+^/K^+^-ATPase optical density than the entire non-malignant areas (0.12 ± 0.031) (*p* < 0.0001). The higher Na^+^/K^+^-ATPase optical density in the non-malignant glands could in part be due to the double cell layer with basal cells lining the epithelial cells in non-malignant glands **(**Fig. 2e**)**, as opposed to PCa glands with only a single layer epithelium without basal cells and characteristic large nuclei with nucleoli (Fig. 2g).

We attempted to compensate for this by estimating the actual Na^+^/K^+^-ATPase optical density per cell profile (Fig. 2f). The Na^+^/K^+^-ATPase optical density per cell profile is 38% (1.38 with 95% CI [1.28; 1.49]) (*p* < 0.0001) higher in non-malignant prostate cells than in PCa cells. Na^+^/K^+^-ATPase optical density per cell profile is 24% (1.24 with 95% CI [1.15; 1.35]) (*p* < 0.0001) higher in non-malignant areas than in malignant areas when estimated on whole tissue basis.

Correlations between cell density, IHC and SUVmax, and ISUP GG are found in Table 3.

**Table 3:** Correlations between immunohistochemistry, cell profile density and ISUP GG and ^82^Rb SUVmax for cancer only (*n* = 82)

		SUVmax	ISUP GG
Cell profile density	Glands	*r* = 0.08 *p* = 0.49	rho = 0.21 *p* = 0.06
	All area	*r* = 0.07 *p* = 0.54	rho = 0.16 *p* = 0.15
Na+/K + -ATPase optical density	Glands	*r* = −0.11 *p* = 0.32	rho = −0.03 *p* = 0.81
	All area	*r* = 0.01 *p* = 0.91	rho = −0.01 *p* = 0.936
Na+/K+-ATPase optical density per cell profile	Glands	*r* = −0.11 *p* = 0.32	rho = −0.17 *p* = 0.12
	All area	*r* = −0.02 *p* = 0.89	rho = −0.12 *p* = 0.30

## Discussion

The main results of the present study are as follows: TBF is the superior predictor of PCa aggressiveness compared to lowest ADC value, and TBF can provide valuable information in separation of clinically significant from insignificant PCa.

### Tumour blood flow predicts cancer aggressiveness

Quantitative TBF can be estimated on different modalities, ideally with gold standard [^15^O]-H_2_O PET, but other flow tracers for PET and MRI with quantitative DCE analysis [2], arterial spin labelling [34], or hyperpolarization [35] can perform qualified estimates. TBF measurement using semiquantitative SUV of ^82^Rb is a very simple analysis, which does not require advanced software and can hence be performed by any department equipped with a PET scanner and an ^82^Rb generator.

**Fig. 3:**
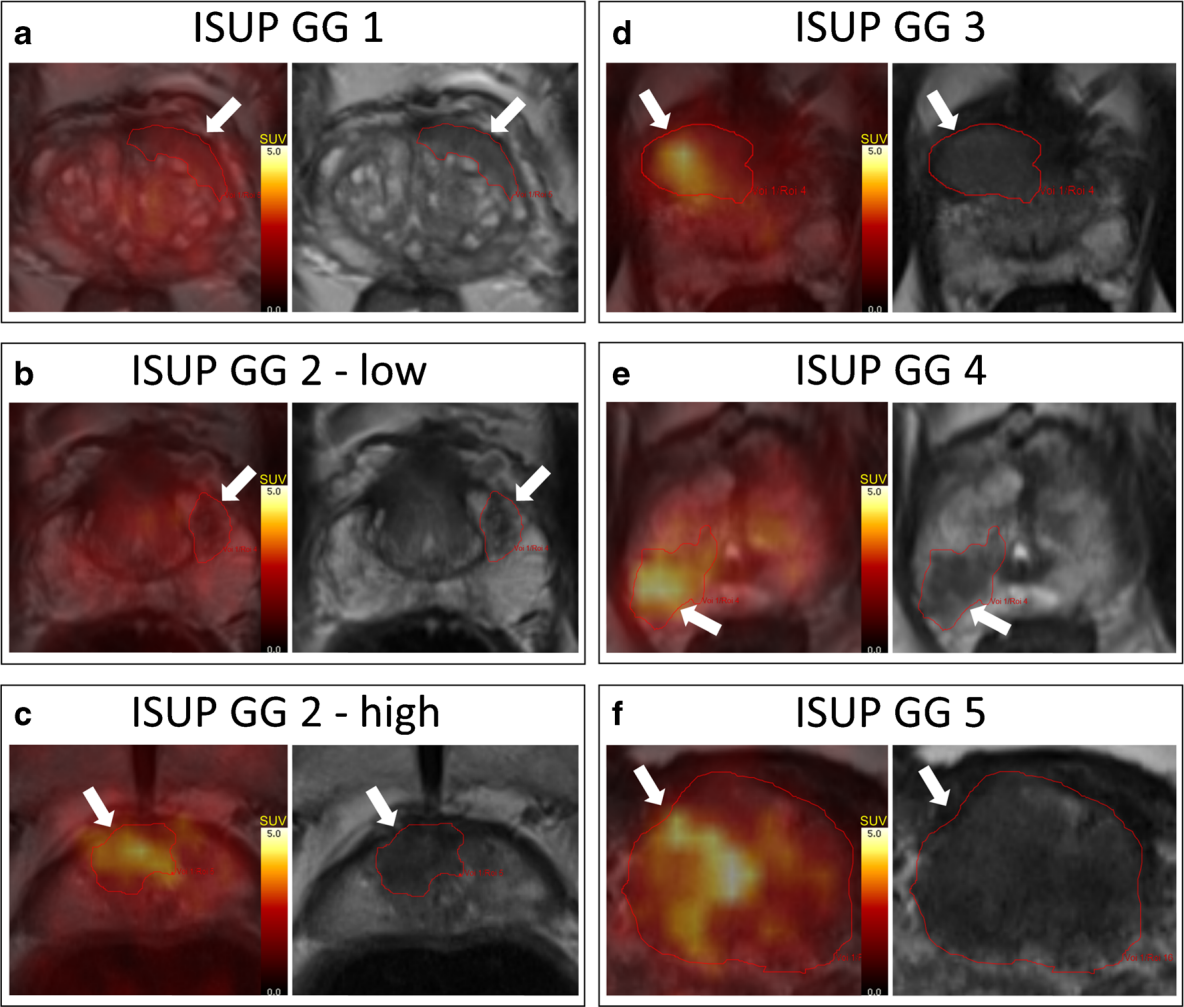
^82^Rb TBF for different ISUP GG. Transaxial PET/MRI fused images and t2w MRI for ISUP GG-1-5, including GG-2 tumours with both high- and low blood flow, illustrating the general increase in tumour blood flow through the spectrum of prostate cancer aggressiveness

In the present study, TBF measurements correlate moderately with PCa aggressiveness. This is consistent with our previous report that TBF is a predictor of GG [10]. Correction for tumour volume and lowest ADC did not change the linear relationship notably, which means that TBF is an independent predictor. Figure 3 demonstrates the TBF of tumours of different ISUP GG, illustrating the general increase in TBF in aggressive tumours compared with that of low risk tumours.

As seen on Fig. 1b, SUVmax for most non-inflammatory benign lesions and ISUP GG-1 tumours is below 3.0 **(**Fig. 3a**)**, and almost all ISUP GG-3-5 tumours are above 3.5 (Fig. 3d, e, and f). On the contrary, TBF of ISUP GG-2 is spread out and almost equally divided over and under SUVmax 3.5 (Fig. 3b and c). Previous studies have shown that the percentage of Gleason 4 is a major determinant of cancer aggressiveness and patient outcome and that a further subdivision based on this percentage is relevant [32, 33]. It was disappointing, however, that the variation of ^82^Rb TBF in ISUP GG-2 was not a good reflection of the percentage of Gleason 4. The clinical relevance of this intragroup variation of ^82^Rb TBF amongst ISUP GG-2 patients can only be determined from follow-up of the patients. The high TBF ISUP GG-2 seems to be more metabolically active and might need more frequent follow-up during active surveillance.

The calculated sensitivities and specificities of TBF for detection of clinically significant PCa were performed with ROC analysis, and the best-balanced SUVmax thresholds were identified. The data show that TBF can provide valuable information in separation of clinically significant from insignificant PCa.

The studied tumours generally display a very heterogeneous blood flow, which is not surprising as prostate tumours are heterogeneous in many aspects. Figure 3f illustrates an example of a large heterogeneous ISUP GG-5 tumour with both high and low blood flow areas, which was actually missed on TRUS biopsies due to its location in the prostate basis. The ISUP GG-3 tumour in Fig. 3d was missed as many as five times on TRUS biopsies because of its anterior location. SUVmax correlated best with ISUP GG (Table 1), which probably means that only a small area of high blood flow is needed to indicate a more aggressive tumour. In lesions with a small area of high blood flow, the SUVmean is not increased notably compared with a universal low flow tumour.

A disadvantage of ^82^Rb is the relative underestimation of high flow values compared with [^15^O]-H_2_O PET, caused by the incomplete extraction of ^82^Rb from the blood [23]. The tissue specificity is another disadvantage of this method for TBF quantification, since both areas with benign prostatic hyperplasia and inflammation can have equally high ^82^Rb uptake as PCa. Hence, it is not possible to conclude solely from the ^82^Rb PET/CT whether a specific area represents cancer. The TBF measurement needs to be part of a multiparametric procedure and an algorithm along with MRI for anatomical and functional mapping of the prostate and other physiological parameters such as diffusion and potentially hypoxia.

### Lowest ADC value and DCE as predictors of cancer aggressiveness

An inverse relationship between lowest ADC value and ISUP GG is established in peripheral zone cancers [36, 37], and therefore, the lowest ADC value is used clinically for pinpointing the target of MRI-guided biopsies. However, our data show that lowest ADC value is inferior to TBF in predicting ISUP GG.

Our data, on the other hand, indicates that DCE could have a similar ability to differentiate significant from non-significant cancer as ^82^Rb TBF; as the patients scored DCE negative, all had benign or relatively low risk cancer (Fig. 1b, orange triangles). However, all other patients in Fig. 1b were scored DCE positive, which illustrates that this qualitative eyeballing approach of gadolinium curves misses substantial information about blood flow physiology gathered by actual TBF quantification. Similarly, both Hötker et al. and Cristel et al. found that pharmacokinetic quantitation of TBF improved the positive predictive value of mpMRI, and thereby provided valuable information in deciding which patients to biopsy [1, 2]. Hence, it is possible that the DCE parameter is removed from the prostate protocols, partially because it is not fully utilized.

### Na+/K+-ATPase optical density in cancer versus normal prostate tissue

The current study analysed Na^+^/K^+^-ATPase expression by IHC, which provides some insights into human prostate tumour biology. Na^+^/K^+^-ATPase optical density per area is 58% higher in cancer areas than in non-malignant areas, which is explained by the 96% higher cell density in cancer areas than in non-malignant areas. We found that non-malignant prostate cells have 24–38% higher expression of Na^+^/K^+^-ATPase per cell than PCa cells in line with a previous animal study [38].

Studies have showed a loss of α1-Na^+^/K^+^-ATPase with increasing tumour grade in dog PCa [38], and in human lung adenocarcinoma [39], bladder cancer [40], colorectal cancer [41], and renal cell carcinoma [42]. The Na^+^/K^+^-ATPase expression of breast cancer on the other hand is higher than in non-malignant breast tissue and associated with worse outcome [43]. In line with Mobasheri et al. [38], we found that the Na^+^/K^+^-ATPase density per cell was significantly lower in higher grade tumours (ISUP GG ≥ 2) (6.4E-5 [6.0E-5; 6.9E-5]) than in low grade tumours (ISUP GG-1) (7.7E-5 [6.5E-5; 8.9E-5]) (*p* = 0.02). Additionally, there was a trend on Spearman’s rank correlation towards a loss of Na^+^/K^+^-ATPase with increasing ISUP GG (rho = − 0.17, *p* = 0.12). We have seen examples of relatively dedifferentiated cancer areas with massive loss of Na^+^/K^+^-ATPase (Fig. 2h); however, it is not a consistent linear decrease with higher ISUP GG as none of the ISUP GG-5 biopsies lost Na^+^/K^+^-ATPase expression in this scale (Fig. 2i and j).

We found a near significant positive correlation between cell density and GG (rho = 0.21, *p* = 0.06), indicating a more compact architecture as the glands become increasingly dedifferentiated and lose their glandular structure.

Based on our data, it seems that Na^+^/K^+^-ATPase optical density and cell density in PCa play very little role in determining the ^82^Rb uptake in the tumour. However, it should be noted that, even though Na^+^/K^+^-ATPase density is not the major limitation of tumour ^82^Rb uptake, our analysis does not measure the actual Na^+^/K^+^-ATPase activity. Azizan et al. showed that different mutations in adrenal adenomas resulted in altered Na^+^/K^+^-ATPase function [44], and from our analysis, this cannot be ruled out in prostate tumours. In conclusion, our findings are compatible with the general use of ^82^Rb PET for measuring perfusion and with our previous results, showing that ^82^Rb uptake relates closely to actual prostate TBF [10].

### Future perspectives

Our data suggest that TBF quantitation could provide valuable clinical information at least in selected patient groups, for example, in equivocal MRI lesions and during active surveillance for repeated assessment of tumour metabolic activity. It would also be interesting to investigate whether the change in blood flow as response to medical or focal therapy is predictive of long-term effect. As there are different ways of measuring TBF, it would be obvious to extract further information from the DCE MRI by quantitative analysis, but an approach with either ^82^Rb or ^15^O-H_2_O PET as an add on examination to MRI appears to be feasible as well.

The primary tumour prostate-specific membrane antigen (PSMA) expression was also reported to correlate with the dominant intraprostatic lesion ISUP GG in a number of studies, however with substantial overlap between groups [45–49]. A direct comparison between ^82^Rb and PSMA PET is needed to determine which physiologic parameter is closest related to the tumour aggressiveness and how they relate to each other.

## Conclusion

TBF is an independent predictor of PCa aggressiveness. TBF quantification rather than qualitative perfusion analysis may provide valuable additional clinical information at least in selected patient groups for separating significant from insignificant PCa.

PCa Na^+^/K^+^-ATPase expression was lower than in non-malignant prostate cells. Neither cell nor Na^+^/K^+^-ATPase optical density determined the ^82^Rb uptake in prostate tumours.

## Electronic supplementary material

ESM 1 (DOCX 12 kb)

ESM 2 (DOCX 12 kb)

## Data Availability

The data supporting the conclusions of the present paper is included.
