# Supplementary material for: Tumour blood flow for prediction of human prostate cancer aggressiveness: a study with Rubidium-82 PET, MRI and Na+/K+-ATPase-density
Source: Eur J Nucl Med Mol Imaging. 2020 Aug 18;48(2):532–42. doi: 10.1007/s00259-020-04998-2 (PMC7835182; doi:10.1007/s00259-020-04998-2)
Supplement: Supplementary file 1 — (DOCX 12 kb) [file 259_2020_4998_MOESM1_ESM.docx]

| Measure | Cut-off | All lesions (n=124) | | | Inflammatory lesions excluded (n=109) | | |
| --- | --- | --- | --- | --- | --- | --- | --- |
|  |  | AUC | Sensitivity | Specificity | AUC | Sensitivity | Specificity |
| SUVmax | 2.92 | 0.79 | 96% | 59% | 0.87 | 96% | 71% |
| SUVmean | 1.94 | 0.73 | 86% | 56% | 0.80 | 87% | 63% |
| SUVpeak | 2.39 | 0.74 | 81% | 56% | 0.80 | 81% | 66% |
| Lowest ADC | 752 | 0.64 | 73% | 54% | 0.64 | 72% | 56% |

**Supplementary table 1.** Results of ROC analysis for separation of ISUP GG>1 from GG1 and benign lesions for both all lesions and for inflammatory lesions excluded for SUVmax, SUVmean, SUVpeak and lowest ADC value, respectively.
