# Supplementary material for: Tumour blood flow for prediction of human prostate cancer aggressiveness: a study with Rubidium-82 PET, MRI and Na+/K+-ATPase-density
Source: Eur J Nucl Med Mol Imaging. 2020 Aug 18;48(2):532–42. doi: 10.1007/s00259-020-04998-2 (PMC7835182; doi:10.1007/s00259-020-04998-2)
Supplement: Supplementary file 2 — (DOCX 12 kb) [file 259_2020_4998_MOESM2_ESM.docx]

| Measure | Cut-off | All lesions (n=124) | | | Inflammatory lesions excluded (n=109) | | |
| --- | --- | --- | --- | --- | --- | --- | --- |
|  |  | AUC | Sensitivity | Specificity | AUC | Sensitivity | Specificity |
| SUVmax | 3.48 | 0.82 | 95% | 63% | 0.85 | 95% | 66% |
| SUVmean | 2.18 | 0.81 | 95% | 58% | 0.83 | 95% | 60% |
| SUVpeak | 2.65 | 0.81 | 95% | 64% | 0.83 | 95% | 67% |
| Lowest ADC | 668 | 0.56 | 60% | 62% | 0.55 | 60% | 61% |

**Supplementary table 2.** Results of ROC analysis for separation of ISUP GG>2 from GG1-2 and benign lesions for both SUVmax, SUVmean, SUVpeak and lowest ADC value for both all lesions and for inflammatory lesions excluded.
